# Case report: Triple whammy: Synchronous radiotherapy induced glioblastoma multiforme and papillary thyroid cancer following nasopharyngeal carcinoma

**DOI:** 10.3389/fonc.2022.1012395

**Published:** 2022-11-14

**Authors:** Kamalanathan Palaniandy, Zalikha Kamarudin, Yin Ping Wong, Shahizon Azura Mohamed Mukari, Winnie Xiao Hui Jiau, Azizi Abu Bakar

**Affiliations:** ^1^ Division of Neurosurgery, Faculty of Medicine, Universiti Kebangsaan Malaysia, Cheras, Malaysia; ^2^ Department of Pathology, Faculty of Medicine, Universiti Kebangsaan Malaysia, Kuala Lumpur, Malaysia; ^3^ Department of Radiology, Faculty of Medicine, Universiti Kebangsaan Malaysia, Kuala Lumpur, Malaysia; ^4^ Department of Rehabilitation Medicine, Hospital Rehabilitasi Cheras, Kuala Lumpur, Malaysia

**Keywords:** synchronous radiotherapy induced malignancy, glioblastoma multiforme, papillary thyroid carcinoma, nasopharyngeal carcinoma, radiotherapy - adverse effects

## Abstract

Secondary malignancies following radiotherapy are well documented, with an estimated incidence of 5%. These may manifest as carcinomas, gliomas, or sarcomas within the previous radiation field. Glioblastoma multiforme following radiotherapy for nasopharyngeal carcinoma is an uncommon occurrence and carries a poor prognosis, whereas papillary thyroid carcinoma following radiotherapy is well documented, though the exact incidence is not well documented. The occurrence of synchronous radiotherapy-induced malignancy over both sites has not been described in the literature before. We describe a middle-aged gentleman diagnosed with glioblastoma multiforme and papillary thyroid carcinoma 6 years after radiotherapy for nasopharyngeal carcinoma. Though our case is the first reported case of a synchronous tumour of its nature, it is likely that such cases are under-reported. Long-term vigilance for loco-regional radiotherapy-induced secondary malignancies is a must, and the presence of a second distinct secondary malignancy must be entertained.

## Introduction

The first case of radiation-induced papillary thyroid carcinoma (PTC) was reported in 1950 by Duffy et al., and glioblastoma multiforme (GBM) was reported in 1978 by Kleriga et al. (1, 2) There is a reported incidence of 17%–19% secondary tumours following successful treatment of primary tumours; the incidence of secondary malignancies following radiotherapy contributes 5% (3). Synchronous tumours refer to cases in which the second primary cancer is diagnosed within 6 months of primary cancer. When one looks into the incidence of synchronous/metachronous primary malignant gliomas and non-central nervous system primary tumours, the overall incidence is 13.1%, of which 5.5% involve the thyroid gland (4). In a publication by Hamzah et al., 9 out of 1,547 patients with primary malignant glioma had cancers of the thyroid gland. This allows us to deduce that the incidence of synchronous/metachronous primary thyroid gland malignancies in the presence of malignant gliomas is less than 0.6%. A search of the literature did not reveal any reports of synchronous radiotherapy-induced malignancy (SRIM) involving malignant gliomas and non-central nervous system tumours. As a matter of fact, there is a paucity of data on SRIM in general. We discuss a case of rapidly symptomatic radiation-induced GBM and papillary thyroid carcinoma in a 42-year-old man who had completed a course of radiation therapy for the head and neck region for nasopharyngeal carcinoma 6 years prior.

## Case report

**Figure 1:**
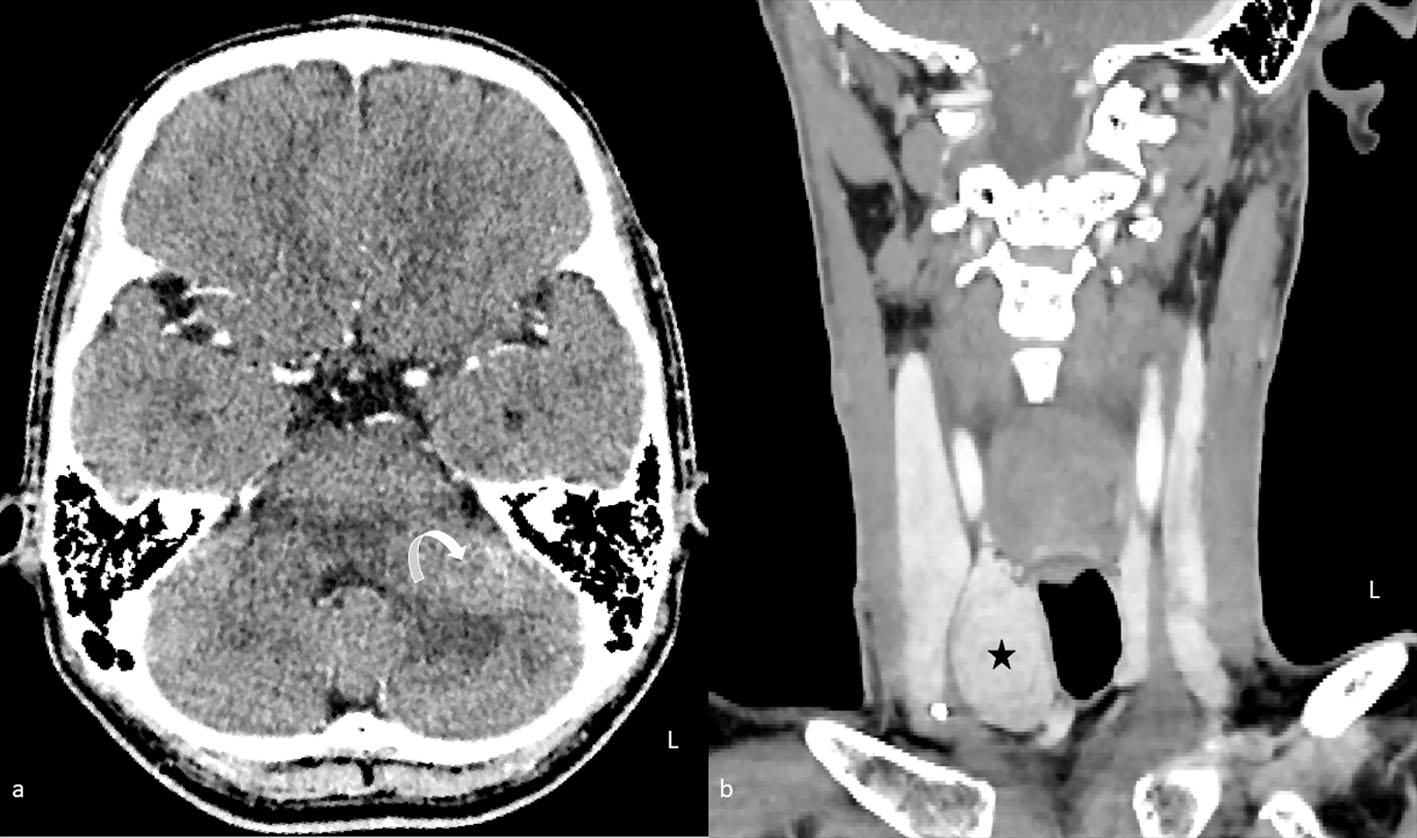
Contrast-enhanced computed tomography of the brain **(A)** in axial plane and neck **(B)** in coronal plane. An enhancing lesion in the left middle cerebellar peduncle is causing mass effect with mild effacement of the fourth ventricle (curved arrow). A right thyroid nodule is also seen synchronously (star).

**Figure 2:**
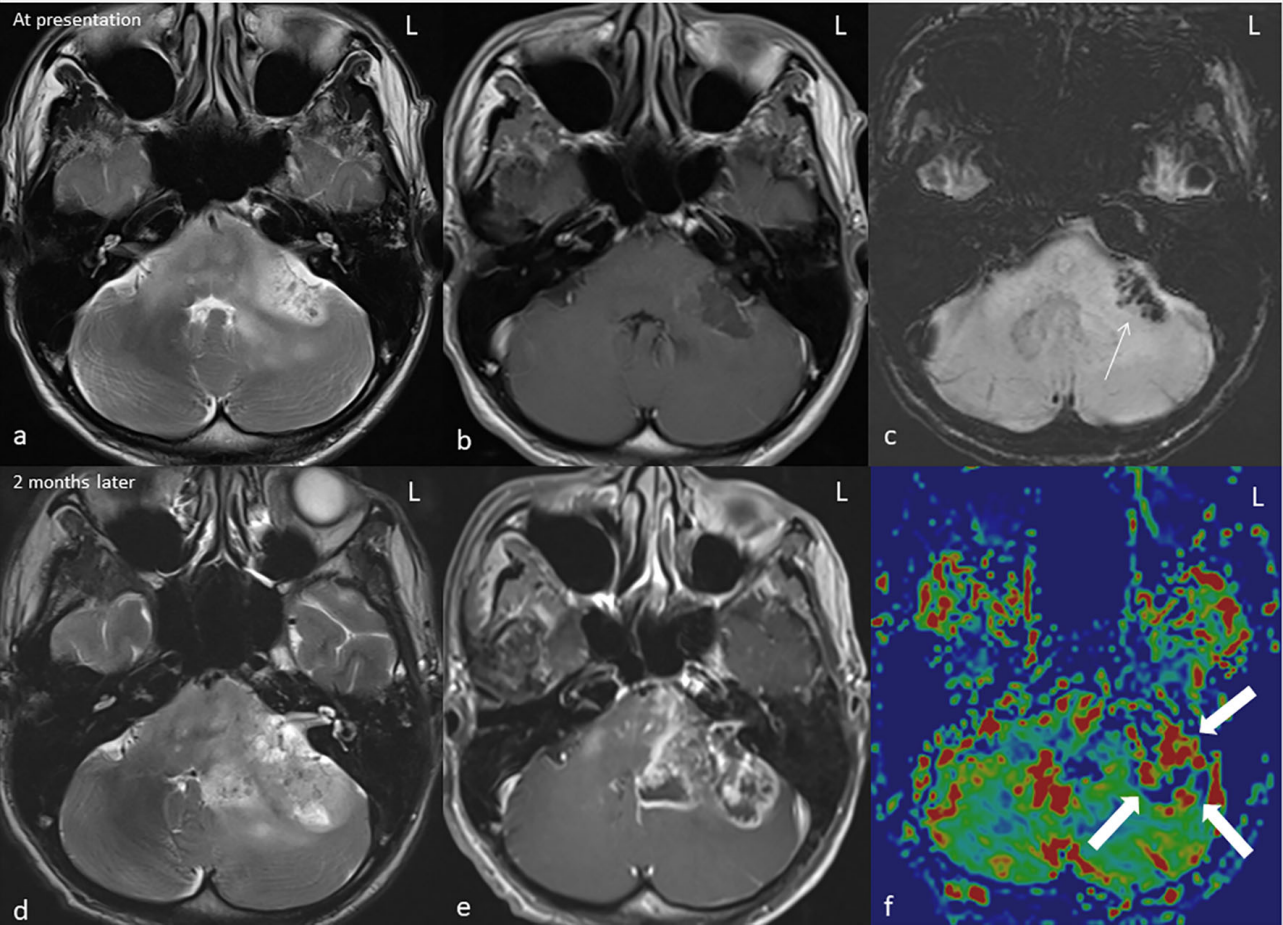
MRI* of the brain in axial T2W** **(A, D)**, T1W*** post gadolinium **(B, E)**, SWI**** **(C)**, and rCBV***** colour map **(F)** at presentation (top row) and at 2 months (bottom row). The left middle cerebellar peduncle mass demonstrates a heterogeneous hyperintense signal on T2 with minimal peripheral enhancement on T1 post-contrast. The hypointensity on SWI*** indicates presence of hemosiderin (arrow). The lesion significantly increases in size on the follow-up MRI (**C, D**) causing significant mass effect; the enhancement is more heterogeneous with irregular rim enhancement and central necrosis. Increase in rCBV at the enhancing region of the rCBV colour map indicates a high-grade tumour (block arrow). *Magnetic resonance imaging, **T2-weighted, ***T2-weighted, ****susceptibility-weighted imaging, and *****relative cerebral blood volume.

A 42-year-old man was diagnosed with nasopharyngeal carcinoma (NPC) 6 years ago and underwent radiation therapy. Subsequent annual clinical surveillance did not reveal a recurrence of the tumour. His last annual computed tomography (CT) of his brain was 3 years prior, and there was no recurrence or new lesions. He presented 6 years later with an unsteady gait and facial numbness for 2 weeks, as well as slurring of his speech and vomiting. Examination revealed left facial palsy upper motor neuron-type House–Brackmann grade III with positive cerebellar signs on the left. The findings from examinations of the other systems were essentially normal. CT scan of the brain revealed an intra-axial lesion in the left cerebellum with an incidental finding of a right thyroid nodule (**Figure 1**). Magnetic resonance imaging (MRI) revealed a lobulated lesion in the left middle cerebellar peduncle extending into the pons (**Figures 2A–C**). The differential diagnosis of radiation necrosis or a high-grade glioma (radiation related given its location) was given. He was given dexamethasone 2 mg twice a day.

**Figure 3:**
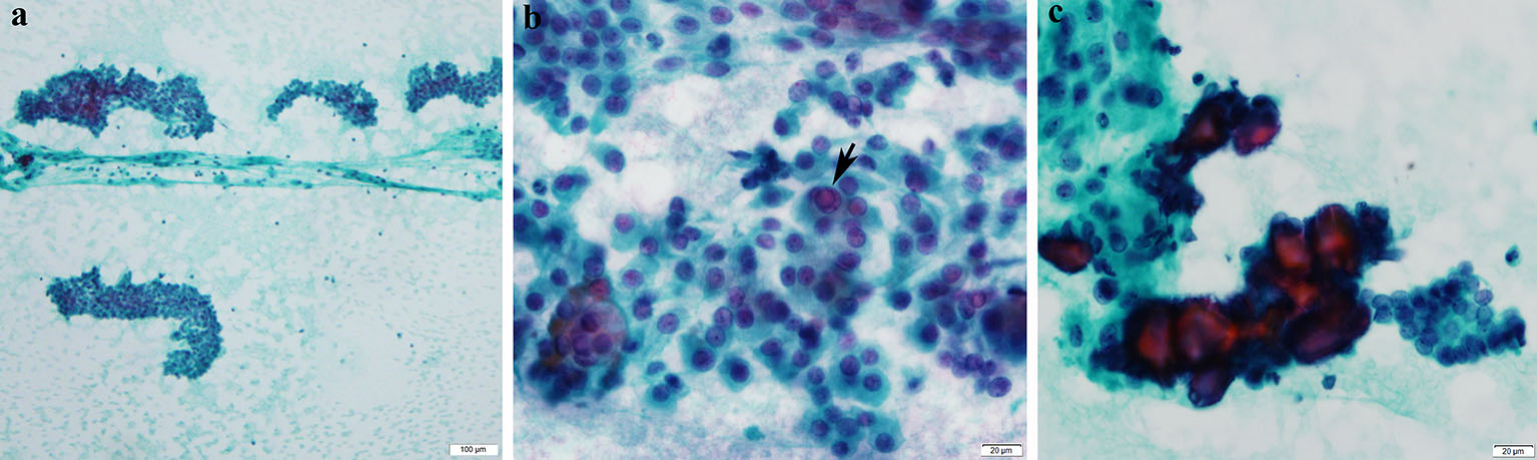
Cytological features of papillary thyroid carcinoma. **(A)** Cellular smears with loose cohesive clusters of malignant cells (Pap*, ×100). **(B)** The malignant cells display enlarged overlapping nuclei with irregular contours and fine powdery chromatin. Occasional intranuclear inclusions (arrow) and grooves are observed (Pap, ×600). **(c)** Psammoma bodies are also noted (Pap, ×600). *Papanicolaou stain.

Unfortunately, his clinical condition worsened, and he progressed to develop gross left lower limb weakness and left bulbar palsy. A follow-up MRI 2 months later revealed enlarging left cerebellar/pons mass with central necrosis (**Figures 2D–F**). An increase in relative cerebral blood volume (rCBV) was noted on the rCBV colour map of MR perfusion of the enhancing component, indicating neoangiogenesis. This was suggestive of a high-grade tumour as opposed to radiation necrosis. Ultrasound-guided, fine-needle aspiration cytology of the enlarged thyroid gland confirmed the presence of papillary thyroid carcinoma (**Figure 3**).

**Figure 4:**
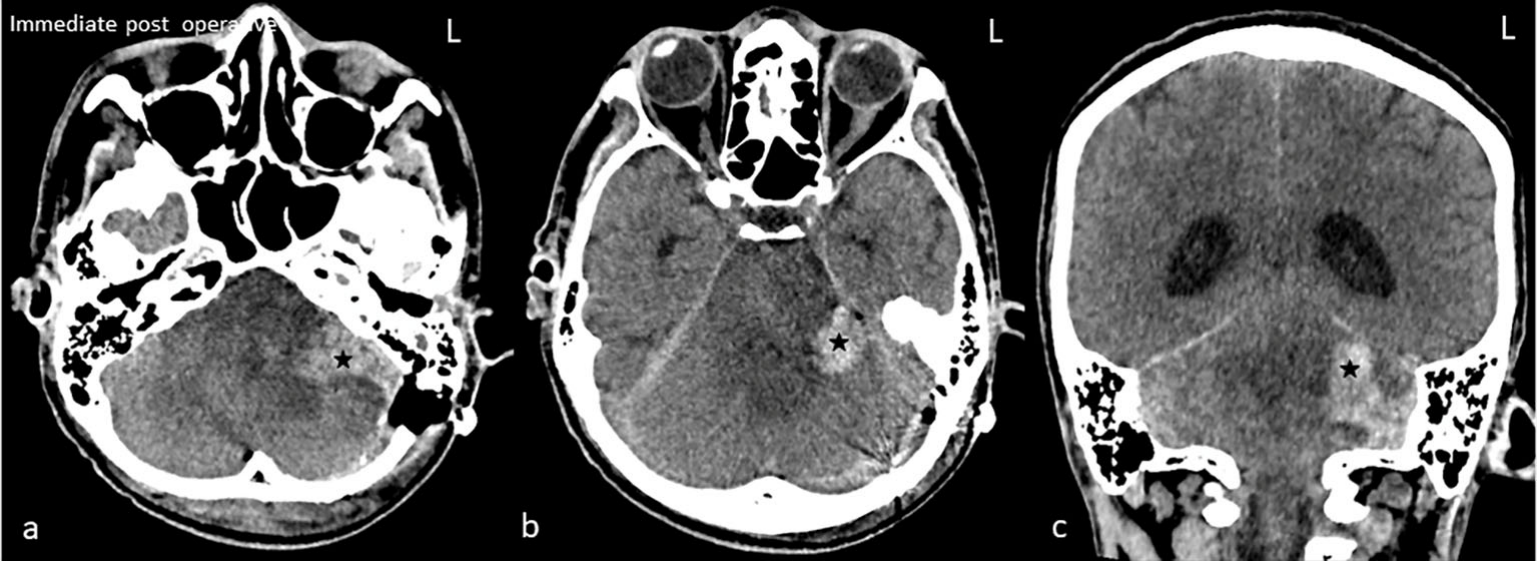
Immediate post-op plain computed tomography of the brain in axial **(A, B)** and coronal **(C)**. Hyperdense hematoma (star) at the left cerebellopontine angle is seen. The fourth ventricle is obliterated with resultant hydrocephalus.

**Figure 5:**
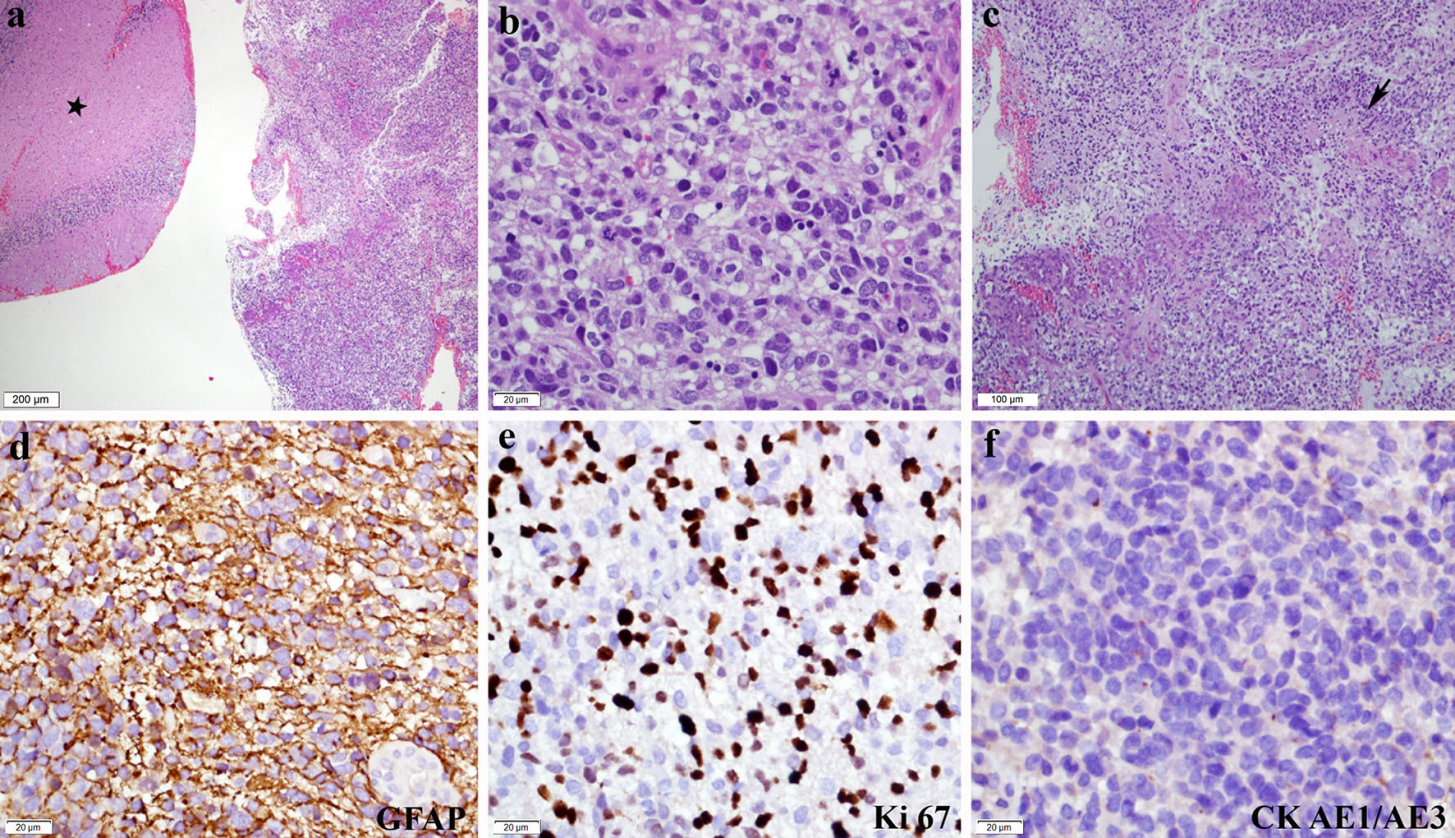
Histopathological and immunohistochemistry features of glioblastoma. **(A)** Unremarkable cerebellar tissue (asterisk) with adjacent tumour tissue (H&E*, ×40). **(B)** Composed of diffuse sheets of malignant cells exhibiting pleomorphic hyperchromatic nuclei with inconspicuous nucleoli and moderate cytoplasm. Mitotic figures are frequently seen, including the atypical form (H&E, ×400). **(C)** Microvascular proliferation and geographical necrosis (arrow) are also noted (H&E, ×100). Immunohistochemically, the malignant cells showed **(D)** diffuse GFAP** positivity (GFAP, ×400). **(E)** Ki 67 proliferative index is high (Ki67, ×400), while **(F)** CK*** AE1/AE3 is negative (CK AE1/AE3, × 400). *Haematoxylin and eosin stain, **glial fibrillary acidic protein, and ***cytokeratin stain.

He became stuporous with a Glasgow Coma score of 11/15 (E2 V3 M6) a week after the follow-up MRI, with normal vital signs. Upon discussion with and consent by his immediate kin, he underwent left retrosigmoid suboccipital craniotomy and debulking of the tumour. Intraoperatively, the tumour was granular with areas of necrosis and thrombosis from the left cerebellar peduncle extending into the superior aspect of the pons. His immediate post-op CT showed a small haematoma at the left cerebellopontine angle with no mass effect and hydrocephalus (**Figure 4**). Histopathological examination confirmed the presence of glioblastoma multiforme, World Health Organization grade IV (**Figure 5**). Despite regaining consciousness, he was subjected to palliative care, as his Karnofsky score was less than 70, and he had already undergone focal radiotherapy at the same region. He succumbed to illness 2 weeks later.

## Discussion

The carcinogenic potential of ionizing radiation first came to be described approximately 7 years after Wilhelm Roentgen proved the presence of electromagnetic radiation of a specific wavelength termed “X-ray” in 1895. This mutation results from a broad spectrum of deoxyribonucleic acid (DNA) lesions including damage to nucleotide bases, cross-linking, and DNA single- and double-strand breaks (DSBs) (5). Misrepaired clustered DSBs along with the inactivation of tumour suppressor genes by loss of heterozygosity result in mutagenic DNA lesions susceptible to the formation of SRIM (5).

This patient underwent conventional radiotherapy. As opposed to intensity-modulated radiotherapy or image-guided radiation therapy, increased radiation exposure to normal adjacent tissue is to be expected. This in turn places the patient at an increased risk of SRIM. Photon beam, used in conventional radiotherapy, carries a higher risk of SRIM, as its dose deposition is thought to be quasi-exponential as opposed to proton beam, whose dose deposition ends sharply at the end of its range (3).

Diagnosis of radiation-induced malignancies is based on four established criteria, which were first mooted by Cahan et al. in 1948 and subsequently modified by Schrantz and Araoz in 1972 (6, 7), the first of which is that the tumour has to be present in a previously irradiated area. Additionally, the histology of the previous tumour should be distinct from that of the current tumour. The third criterion is that there has to be sufficient latency between exposure to radiation and the onset of the new malignancy, which is usually more than 5 years. The final criterion is the absence of genetic predisposition for the development of cancer, such as seen in the Li–Fraumeni syndrome or retinoblastoma.

In our patient, the previous tumour was NPC, whereas the current diagnoses are PTC and GBM. If one is to take into consideration the initial NPC, these are three different distinct histological diagnoses and anatomical locations. The latency between radiotherapy and diagnosis was 6 years with no clinical evidence to suggest genetic predispositions. The common factor here is the history of radiotherapy to the head and neck for NPC, which exposes the thyroid gland and brain to radiation. As all four criteria were fulfilled, we concluded that both GBM and PTC were of SRIM.

Our patient presented with progressive neurological symptoms; as such, it is only natural that the presence of a neurological pathology is sought. Unfortunately, the diagnosis of GBM was not made in the first instance, as a clear distinction between radiation necrosis and GBM with a necrotic core cannot be made with traditional MRI sequences. The typical conventional MRI findings include an enhancing mass with central necrosis and perilesional vasogenic oedema. The enhancing mass has a margin that is ill-defined, often described as a “spreading wavefront” or mass with central necrosis, described as “soap bubble-like” or “Swiss cheese-like” (8).

Advanced MRI techniques such as diffusion-weighted imaging, magnetic resonance spectroscopy, perfusion-weighted imaging, and positron emission tomography with various radionuclides are being applied in this respect; however, their efficacy and reliability still require further validation (9). MR perfusion has parameters that may help to differentiate radiation necrosis and tumour recurrence or high-grade tumour to some extent, namely, the rCBV, where the latter will show high rCBV and radiation necrosis demonstrates hypoperfusion (8).

This conundrum is further accentuated by the temporality of the term “sufficient latency between radiation exposure”, and the occurrence of SRIM is still not well defined. As for the diagnosis of PTC, it was an incidental finding when we attempted to restage the primary disease, which was NPC. If not for this indication, a CT scan of the neck would not have been performed, and this diagnosis would have been missed.

The occurrence of intracranial SRIM is well-documented yet poorly understood and unpredictable. The most common tumours encountered are gliomas, of which approximately 58% are GBM (10). Treatment options for SRIM-associated GBM are similar to those for *de novo* GBM. The median survival is 10 months with the administration of temozolomide not making any significant difference; ironically, multimodality treatment with the inclusion of radiotherapy appears to increase survival to 18 months (10). Unfortunately, our patient succumbed to his illness less than 2 months after the initial presentation. This was likely due to the advanced presentation of intracranial diseases, where the brainstem was already invaded by the tumour. The surgery in this case was a diagnostic and temporising lifesaving procedure in view of the diagnostic dilemma and rapidly progressive symptoms.

Thyroid cancers following radiation exposure are well-recognised and researched. However, this remains largely so in the domain of radioactive fallouts or disasters and radiation exposure in the paediatric age group, again pertaining to radioactive fallouts and diagnostic radiology. The occurrence of SRIM in the thyroid following radiotherapy remains to be scarce in recent literature. PTC continues to be the most common thyroid malignancy following radiation. The unique observation here is that among the survivors of the Chernobyl disaster, most young children had a solid follicular PTC subtype with an aggressive behaviour and a short latency period, whereas older children had more frequently classical PTC that was less aggressive and was discovered after a long latency period (11). Those undergoing radiotherapies are predominantly adults, and the primary malignancies are often aggressive resulting in reduced survival; thus, the latency may not be reached in these patients. This is coupled with the finding of less aggressive behaviour of the PTC, which may result in a clinically silent PTC as was in our patient. The management of SRIM PTC is similar to that of *de novo* PTC with a similar outcome, notwithstanding the course of the primary disease.

## Conclusion

Our case is possibly the first report of synchronous SRIM that highlights the importance of vigilance. Long-term follow-up for patients with a previous history of radiation is important not only with respect to the primary malignancy but also to be cautious of SRIM. Should an SRIM be diagnosed, the previous radiotherapy plan should be revisited, and the region(s) irradiated should be screened for the possibility of synchronous SRIM.

## Data availability statement

The original contributions presented in the study are included in the article/supplementary material. Further inquiries can be directed to the corresponding author.

## Ethics statement

Ethical review and approval was not required for the study on human participants in accordance with the local legislation and institutional requirements. The patients/participants provided their written informed consent to participate in this study. Written informed consent was obtained from the individual(s) for the publication of any potentially identifiable images or data included in this article.

## Author contributions

All the authors have contributed by means of clinical management, proof reading as well as processing and contributing the histological and radiological diagnosis. All authors contributed to the article and approved the submitted version.

## Conflict of interest

The authors declare that the research was conducted in the absence of any commercial or financial relationships that could be construed as a potential conflict of interest.

## Publisher’s note

All claims expressed in this article are solely those of the authors and do not necessarily represent those of their affiliated organizations, or those of the publisher, the editors and the reviewers. Any product that may be evaluated in this article, or claim that may be made by its manufacturer, is not guaranteed or endorsed by the publisher.
